# Managing supraventricular tachyarrhythmia in pregnant patients within the emergency department

**DOI:** 10.3389/fcvm.2024.1517990

**Published:** 2024-12-10

**Authors:** Di Pan, Zhongqing Chen, Haibo Chen

**Affiliations:** ^1^ Department of Cardiology, Shenzhen Second People’s Hospital, The First Affiliated Hospital of Shenzhen University, Shenzhen, China; ^2^ Department of Emergency, Shenzhen Second People's Hospital, The First Affiliated Hospital of Shenzhen University, Shenzhen, China

**Keywords:** tachycardia (SVT), pregnancy, emergency department, arrhythmia treatment, maternal cardiac care

## Abstract

**Background:**

Pregnancy increases the risk of supraventricular tachycardia (SVT) due to physiological changes. This study reviews the management of SVT in pregnant patients in the emergency department (ED).

**Methods:**

We retrospectively analyzed 15 pregnant patients with SVT treated at Shenzhen Second People's Hospital ED from 2015 to 2023. Treatments included vagal nerve stimulation, pharmacotherapy, esophageal pacing, cardioversion, and radiofrequency ablation.

**Results:**

The average patient age was 30.3 years. All presented with palpitations, and none had hemodynamic instability. Treatment success varied: 3 patients reverted spontaneously, 5 responded to vagal stimulation, and 4 to esophageal pacing. One required verapamil, and another responded to labetalol after failing vagal and pacing treatments.

**Conclusion:**

When managing SVT during pregnancy, it is important to consider the patient's stability, the stage of pregnancy, and the safety of medications. For unstable patients, electrical cardioversion is the preferred option; for stable patients, vagus nerve stimulation (VNS) or other alternative treatments, such as adenosine, should be considered.

## Introduction

Arrhythmia incidence is increasing among pregnant women, particularly those with structural heart disease. While most arrhythmias pose no risk to pregnant women or their fetuses, expectant mothers should actively address severe cases. Supraventricular tachycardia (SVT), which includes atrioventricular nodal reentrant tachycardia (AVNRT) and atrioventricular reentrant tachycardia (AVRT), is common among pregnant women (1, 2).

Abnormal heart rhythms in pregnant women result from a complex interplay of physiological, hormonal, and cardiac structural changes. Initially, increased cardiac workload may modify the heart's electrophysiological properties, potentially leading to arrhythmias. In pregnancy, the heart's output and blood volume rise substantially (typically by 30%–50%) to meet the needs of both the fetus and the mother's body. Additionally, the mother's resting heart rate increases by 10–20 beats per minute to handle the elevated blood volume and cardiac output, placing an additional burden on the maternal heart (3–5). Secondly, pregnancy brings significant hormonal changes, including elevated estrogen and progesterone levels. These fluctuations can affect the heart's electrical properties and vascular tone, potentially leading to arrhythmias (6, 7). Concurrently, the autonomic nervous system experiences alterations during pregnancy, marked by decreased parasympathetic activity and increased sympathetic activity. Heightened sympathetic nervous system activity may lead to abnormal automaticity, reentry, or triggering events (8, 9). Additionally, electrolyte imbalances and an elevated basal metabolic rate can affect the heart's normal electrical activity (10). This article investigates the management of supraventricular tachycardia in pregnant women within the emergency obstetric unit of a major teaching hospital.

## Methods

We retrospectively analyzed electronic medical records of pregnant women diagnosed with supraventricular tachycardia (SVT) at Shenzhen Second People's Hospital Emergency Department from January 1, 2015, to January 1, 2023. Treatment options include vagal nerve stimulation, pharmacotherapy, esophageal pacing, cardioversion, and radiofrequency ablation. Inclusion criteria:Pregnant women aged between 18 and 45 years.Diagnosis of supraventricular tachycardia (SVT) confirmed by electrocardiogram (ECG), defined as regular tachycardia with an atrial rate exceeding 100 beats per minute, with normal P wave and QRS morphology.

Classification of gestational stages: early (1–12 weeks), mid (13–27 weeks), late (28–40 weeks). Exclusion criteria: Patients with structural heart disease or other serious comorbidities. Patients using medications during pregnancy that may affect cardiac function. Patients who refuse to participate in the study or are unable to provide informed consent. The study was approved by the Ethics Committee of Shenzhen Second People's Hospital, and all patients signed an informed consent form.

## Results

From January 1, 2015, to January 1, 2023, the Emergency Department at Shenzhen Second People's Hospital handled 490 cases of supraventricular tachycardia (SVT), including 15 cases related to pregnancy. The baseline information of the patients is shown in Table 1. The average age of these patients was 30.3 ± 4.4 years. Among them, 6 cases occurred during mid-pregnancy, 7 during late pregnancy, and 2 had an unknown pregnancy stage.

**Table 1:** Patient characteristics.

Age, years	30.3 ± 4.4
Pregnancy term, wk	26.9 ± 6.0
History of SVT, *n* (%)	7 (47%)
Number of previous pregnancies	0.6 ± 1.11

All patients arrived at the emergency room with palpitations, and their hemodynamic status remained stable. One patient experienced chest tightness, another had an upper respiratory infection, and a third presented with abdominal pain.

Seven patients had a history of previous supraventricular tachycardia. Among them, three SVT patients spontaneously reverted to sinus rhythm without specific treatment, five achieved sinus rhythm following vagus nerve stimulation, and four immediately returned to sinus rhythm after esophageal pacing. Additionally, one patient restored sinus rhythm after receiving verapamil, another responded to esophageal pacing when propranolol was ineffective, and a third patient regained sinus rhythm after taking labetalol despite unresponsiveness to vagus nerve stimulation and esophageal pacing.The interventions applied to each patient and the acute and long-term outcomes are shown in Table 2.

**Table 2:** Applied intervention, and acute and long-term outcomes for each patient.

Patient ID	Applied intervention	Acute outcome	12-month follow-up
1	VNS	Resolved	No recurrence
2	Esophageal pacing	Resolved	Recurred
3	VNS	Resolved	Data not available
4	Esophageal pacing	Resolved	No recurrence
5	Esophageal pacing	Resolved	No recurrence
6	VNS	Resolved	No recurrence
7	Verapamil	Resolved	Recurred
8	Self-recovery	Resolved	No recurrence
9	VNS	Resolved	Data not available
10	Propacetamol	Unresolved	No recurrence
11	Self-recovery	Resolved	Recurred
12	VNS	Resolved	No recurrence
13	Esophageal pacing	Resolved	No recurrence
14	Self-recovery	Resolved	No recurrence
15	VNS	Unresolved	Recurred

VNS, vagus nerve stimulation.

**Figure 1:**
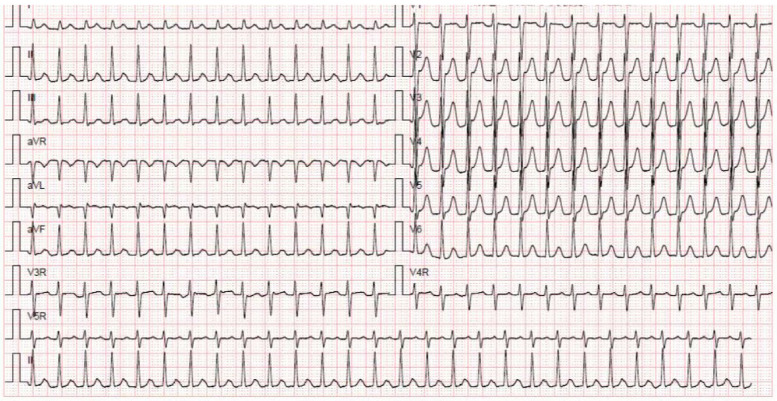
Electrocardiogram of SVT.

We report a case of a 36-year-old pregnant woman who had experienced three pregnancies, including two full-term deliveries and one miscarriage. At 24 weeks into her fourth pregnancy, she experienced her first episode of supraventricular tachycardia (SVT). On November 26, 2017, she sought medical evaluation due to one day of palpitations and a heart rate of 180 beats per minute. An electrocardiogram confirmed SVT (Figure 1), and cardiac ultrasound showed no significant abnormalities. Fetal ultrasound revealed a normal heart rate, regular cardiac rhythm, and normal fetal-placental circulation. Liver and kidney function, electrolytes, troponin I, myoglobin, and creatine kinase isoenzymes were all within normal ranges. When vagal stimulation was ineffective, sinus rhythm was successfully restored using transesophageal pacing. Later in pregnancy, the patient experienced four SVT episodes. The second and third episodes responded to esophageal pacing, while the fourth and fifth episodes reverted after vagal nerve stimulation. A 31-year-old pregnant woman at 21 weeks of gestation was admitted to the gynecology ward on October 4, 2020, due to lower abdominal pain and vaginal bleeding, which raised concern for a threatened miscarriage. During the miscarriage, the patient experienced episodes of supraventricular tachycardia (SVT). Intravenous administration of 5 mg verapamil successfully restored her heart rate to normal sinus rhythm. On the second day after the miscarriage, the patient experienced a recurrence of supraventricular tachycardia. Despite receiving 5 mg of verapamil, the tachycardia persisted. Subsequently, the patient was referred to the cardiology department, where successful esophageal pacing restored normal sinus rhythm. Detailed echocardiography revealed mitral valve prolapse with moderate to severe regurgitation, left atrial enlargement, and normal left ventricular systolic and diastolic function. The patient declined mitral valve replacement surgery. Additional cardiac electrophysiology tests confirmed atrioventricular nodal reentrant tachycardia (AVNRT) involving dual AV nodal pathways (slow-fast type). Recent literature suggests that MVP can predispose patients to atrial arrhythmias due to changes in atrial structure and function (11). In this case, the patient's MVP presented a challenge in selecting the most appropriate treatment for SVT. We opted for a conservative approach initially, with close monitoring and the use of beta-blockers to control heart rate (12). However, given the recurrent nature of the arrhythmia, we eventually proceeded with radiofrequency ablation, a treatment that has been increasingly supported for its efficacy in high-risk populations.

## Discussion

### Acute treatment

With the increasing incidence of supraventricular tachycardia (SVT) during pregnancy, the emergency department needs to respond swiftly and cautiously when managing such cases. Palpitations prompt patients to seek medical evaluation, and the emergency department is often the initial point of assessment for pregnant women with SVT. Optimal management of SVT in emergent scenarios is critical (13, 14). Common approaches include vagal maneuvers, drug therapy, esophageal pacing, electrical cardioversion, and radiofrequency ablation. Direct current cardioversion is generally regarded as safe and effective throughout pregnancy (1, 15, 16). Although it may trigger uterine contractions, it does not compromise fetal blood flow and carries minimal risk of fetal arrhythmias. However, isolated reports of preterm labor exist (17, 18). While fetal impact from electrical cardioversion is minimal, precautions include proper electrode placement away from the fetus, energy levels of 50–100 J, and continuous fetal monitoring during and after the procedure.

For patients without hemodynamic instability, vagus nerve stimulation (VNS) is the recommended treatment (19). This method achieves an efficacy rate of approximately 27.7%. It involves stimulating the tongue root to induce vomiting, applying cold water to the face, performing the Valsalva maneuver, and massaging the carotid sinus. However, the practice of pressing on the eyeballs is being phased out due to the risk of retinal detachment.

Currently, the modified Valsalva maneuver is the most frequently employed technique for vagus nerve stimulation. In the procedure, the patient is placed in a semi-recumbent position and instructed to forcefully exhale into a syringe for 15 s. Afterwards, the patient is quickly moved to a supine position with legs elevated at a 45°angle, maintaining this posture for 45 s. Typically, results become apparent within one minute. Currently, the modified Valsalva manoeuvre is increasingly praised for its higher success rate as an effective method for terminating SVT in pregnant patients. Recent studies, such as that by Lan Q et al. support the superiority of the modified Valsalva manoeuvre over the standard Valsalva manoeuvre in restoring sinus rhythm (20). Additionally, research by Smith GD et al. also emphasises this point. These studies indicate that the modified Valsalva manoeuvre not only improves the success rate but also reduces reliance on antiarrhythmic medications, thereby minimising the risk of adverse drug reactions. However, if the reentrant pathway responsible for the supraventricular tachycardia rhythm does not directly involve the atrioventricular node, vagus nerve stimulation may not achieve the desired outcomes (21, 22).

### Long-term treatment

Currently, there is insufficient clinical research on the effects of anti-supraventricular tachycardia (SVT) drugs on fetuses during pregnancy. All antiarrhythmic medications pass through the placenta, and in theory, they may present risks to both the mother and the fetus. Whenever possible, drug administration should be avoided in early pregnancy, when the risk of congenital malformations is highest. Persistent SVT may necessitate pharmacological treatment. Table 3 summarizes frequently used antiarrhythmic drugs during pregnancy and their potential side effects (1, 23, 24). The selection of antiarrhythmic drugs requires caution to ensure the safety of both mother and child. The study by Fischer AJ et al. emphasizes the specific risks and benefits that need to be considered when using these drugs during pregnancy (25).

**Table 3:** Commonly used antiarrhythmic drugs for treating SVT and their associated side effects.

Drugs	VW Class	FDA category	Placenta permeable	Transfer to breast milk	Adverse effects
Propafenone	IC	C	Yes	Unknown	Absence of human and animal data.
Atenolol	II	D	Yes	Yes	• Growth retardation, • Bradycardia • Hypoglycaemia
Bisoprolol	II	C	Yes	Yes	• Bradycardia • Hypoglycaemia
Metoprolol	II	C	Yes	Yes	• Bradycardia • Hypoglycaemia
Propranolol	II	C	Yes	Yes	• Bradycardia • Hypoglycaemia • Growth retardation
Sotalol	III	B	Yes	Yes	• Bradycardia • Hypoglycaemia
Amiodarone	III	D	Yes	Yes	• Fetal hypothyroidis • Growth retardation • Prematurity • Use only when there is no other option
Diltiazem	IV	C	No	Yes	Absence of human data
Verapamil	IV	C	Yes	Yes	• Bradycardia • Heart block • Intravenously use is may be associated • with a greater risk of hypotension and subsequent fetal hypoperfusion.
Adenosine	NA	C	No	No	No significant adverse effects.

Adenosine, which has a half-life of less than 10 s, is considered safe. Both the European Society of Cardiology (ESC) and the American Heart Association (AHA) guidelines recommend adenosine as the first-line treatment for pregnant women with supraventricular tachycardia (SVT). The initial intravenous dose is 6 mg, with the possibility of administering a 12 mg dose if needed. Common adverse reactions include transient chest pain and breathlessness. Additionally, there have been reports of fetal heart rhythm effects following adenosine use for cardioversion in pregnant women with supraventricular tachycardia (SVT), although some cases did not show any impact on the fetus (26–28). Owing to limited availability of adenosine raw materials in China, numerous hospitals face shortages of adenosine medication.

Extensive clinical experience supports using beta-blockers to treat hypertension during pregnancy. According to the guidelines from the European Society of Cardiology (ESC) and the American Heart Association (AHA), beta-blockers are recommended as a second-line treatment for pregnant women with supraventricular tachycardia (SVT) when adenosine is ineffective or contraindicated. Adverse effects, including fetal bradycardia, growth restriction, and preterm birth, have been thoroughly documented. Metoprolol and propranolol are commonly prescribed medications, whereas atenolol is not recommended due to reduced protein binding, an increased risk of adverse fetal effects, and higher breast milk levels (29–32).

When adenosine and β-blockers are viable treatment options, it is recommended to steer clear of calcium channel blockers due to documented potential adverse effects, such as fetal bradycardia and fetal heart block (33). However, current guidelines continue to endorse STV as the initial treatment for long-term management, with a specific recommendation for verapamil use. However, the use of diltiazem remains controversial due to documented potential lethal and teratogenic effects observed in animal studies (34).

Propafenone is a viable choice for pregnant women with non-organic heart disease who do not respond to the previously mentioned treatments or medications. However, amiodarone, which significantly affects both fetal and maternal thyroid function, is reserved for severe arrhythmias that do not respond to other treatments or pose life-threatening risks (1, 20).

According to the latest guidelines from the European Society of Cardiology (ESC) and the Heart Rhythm Society (HRS), the recommendation level for catheter ablation in the management of supraventricular tachycardia (SVT) is IIA, with a level of evidence rated as C (1). Catheter ablation is suitable for patients with SVT who have significant symptoms and for whom drug treatment has been ineffective, particularly when performed at experienced centres. For patients experiencing severe symptoms or with impaired cardiac function, catheter ablation may be an effective treatment option. The ESC and HRS recommend considering catheter ablation for symptomatic patients with recurrent SVT prior to planned pregnancy, in order to reduce the risks and complexities of treatment during pregnancy.

During the catheter ablation process, it is crucial to avoid the use of fluoroscopy to reduce the potential teratogenic risk to the fetus. Zero-fluoroscopy radiofrequency ablation is an advanced cardiac catheter ablation technique that does not rely on traditional x-ray fluoroscopy but instead utilises technologies such as three-dimensional electroanatomical mapping systems and intracardiac echocardiography to guide the ablation process. A meta-analysis involving over 9,000 patients has shown that zero/minimal fluoroscopy SVT ablation can be performed safely. This indicates that the technique has been proven safe in a large patient population, with no significant complications reported (35). During the catheter ablation process, diaphragm puncture may be required. This is a routine procedure when using fluoroscopic guidance, but it can be more challenging to perform without fluoroscopy. However, scientific data suggest that this procedure can be carried out safely even in the absence of relevant experience (36).

Reentry is the primary electrophysiological mechanism responsible for supraventricular tachycardia (SVT). Esophageal pacing, a non-invasive electrophysiological technique, delivers stimulation pulses to the excitable gap within the reentry circuit. By inducing a new refractory period, it interrupts the forward or backward pathway of the reentry circuit, ultimately stopping the tachycardia. Usually, a cardiac electrophysiology recorder is used, and an esophageal electrode is inserted through the patient's nasal cavity to a position in the esophagus aligned with the left atrium. The ideal atrial pacing site is identified when the esophageal lead detects a tall, biphasic, or multiphasic P wave. Overdrive suppression, underdrive suppression, programmed stimulation, and short burst stimulation are effective methods for stopping tachycardia. While esophageal pacing is common in China, it is rarely reported in Western countries (37).

## Limitations

First, being a retrospective descriptive study, its design has inherent limitations. Second, since this is a single-center study, the research findings may not generalize to all populations. Additionally, the small sample size is influenced by the specific characteristics of the study population. Therefore, more diverse populations and higher-level clinical trials are needed.

## Conclusions

In managing SVT during pregnancy, it is crucial to consider the patient's hemodynamic stability, the stage of pregnancy, and the safety profile of medications. For patients with hemodynamic instability, electrical cardioversion remains the preferred option. For those with stable hemodynamics, initial treatment with vagal maneuvers such as the modified Valsalva maneuver is recommended, with adenosine or other pharmacological interventions considered if vagal maneuvers fail. Our experience highlights the variability in treatment response and the importance of a tailored approach to long-term management, emphasizing the need for close follow-up and readiness to adjust treatment as necessary.

## Data Availability

The raw data supporting the conclusions of this article will be made available by the authors, without undue reservation.

## References

[1] Regitz-Zagrosek V Roos-Hesselink JW Bauersachs J Blomström-Lundqvist C Cífková R De Bonis M 2018 ESC guidelines for the management of cardiovascular diseases during pregnancy. Eur Heart J. (2018) 39(34):3165–241. 10.1093/eurheartj/ehy340 30165544

[2] Owens A Yang J Nie L Lima F Avila C Stergiopoulos K. Neonatal and maternal outcomes in pregnant women with cardiac disease. J Am Heart Assoc. (2018) 7(21):e009395. 10.1161/JAHA.118.009395 30571384 PMC6404206

[3] Clapp JF 3rd Capeless E. Cardiovascular function before, during, and after the first and subsequent pregnancies. Am J Cardiol. (1997) 80(11):1469–73. 10.1016/S0002-9149(97)00738-8 9399724

[4] Adamson DL Nelson-Piercy C. Managing palpitations and arrhythmias during pregnancy. Heart. (2007) 93(12):1630–6. 10.1136/hrt.2006.098822 18003696 PMC2095764

[5] Sanghavi M Rutherford JD. Cardiovascular physiology of pregnancy. Circulation. (2014) 130(12):1003–8. 10.1161/CIRCULATIONAHA.114.009029 25223771

[6] Conrad KP. Emerging role of relaxin in the maternal adaptations to normal pregnancy: implications for preeclampsia. Semin Nephrol. (2011) 31(1):15–32. 10.1016/j.semnephrol.2010.10.003 21266262 PMC3381791

[7] Osol G Ko NL Mandalà M. Plasticity of the maternal vasculature during pregnancy. Annu Rev Physiol. (2019) 81:89–111. 10.1146/annurev-physiol-020518-114435 30742784 PMC6571171

[8] Shen MJ Zipes DP. Role of the autonomic nervous system in modulating cardiac arrhythmias. Circ Res. (2014) 114(6):1004–21. 10.1161/CIRCRESAHA.113.302549 24625726

[9] Vaseghi M Shivkumar K. The role of the autonomic nervous system in sudden cardiac death. Prog Cardiovasc Dis. (2008) 50(6):404–19. 10.1016/j.pcad.2008.01.003 18474284 PMC2752648

[10] Robinson VM Di Diego JM Bowes MT Kowey PR Antzelevitch C Venetucci L. Increased susceptibility to ventricular arrhythmia at low-normal and moderately low levels of extracellular potassium in catecholaminergic polymorphic ventricular tachycardia. Heart Rhythm. (2022) 19(8):1389–91. 10.1016/j.hrthm.2022.04.005 35429650

[11] Nkomo VT Gardin JM Skelton TN Gottdiener JS Scott CG Enriquez-Sarano M. Burden of valvular heart diseases: a population-based study. Lancet. (2006) 368(9540):1005–11. 10.1016/S0140-6736(06)69208-8 16980116

[12] Rahimtoola SH. The natural history of mitral stenosis. Am Heart J. (1978) 95(3):417–23.636979

[13] Tamirisa KP Oliveros E Paulraj S Mares AC Volgman AS. An overview of arrhythmias in pregnancy. Methodist Debakey Cardiovasc J. (2024) 20(2):36–50. 10.14797/mdcvj.1325 38495654 PMC10941715

[14] Saricam E Mutlu MF Ozkan M Barindik N. The treatment for maternal supraventricular tachyarrhythmia in pregnant patients in ED practice. Am J Emerg Med. (2016) 34(8):1702–4. 10.1016/j.ajem.2016.05.049 27241567

[15] Peterson AA Arendt KW Sharpe EE. Management of supraventricular tachycardia in pregnancy. Pain Med. (2020) 21(2):426–428. 10.1093/pm/pnz330 31845980

[16] Tromp CH Nanne AC Pernet PJ Tukkie R Bolte AC. Electrical cardioversion during pregnancy: safe or not? Neth Heart J. (2011) 19(3):134–6. 10.1007/s12471-011-0077-5 21475392 PMC3047673

[17] Hornung TS Bernard EJ Celermajer DS Jaeggi E Howman-Giles RB Chard RB Right ventricular dysfunction in congenitally corrected transposition of the great arteries. Am J Cardiol. (1999) 84(9):1116–9; A10. 10.1016/S0002-9149(99)00516-0 10569681

[18] Blake MJ Martin A Manktelow BN Armstrong C Halligan AW Panerai RB Changes in baroreceptor sensitivity for heart rate during normotensive pregnancy and the puerperium. Clin Sci. (2000) 98(3):259–68. 10.1042/cs0980259 10677383

[19] Enriquez AD Economy KE Tedrow UB. Contemporary management of arrhythmias during pregnancy. Circ Arrhythm Electrophysiol. (2014) 7(5):961–7. 10.1161/CIRCEP.114.001517 25336366

[20] Lan Q Han B Wu F Peng Y Zhang Z. Modified Valsalva maneuver for treatment of supraventricular tachycardias: a meta-analysis. Am J Emerg Med. (2021) 50:507–12. 10.1016/j.ajem.2021.08.067 34536723

[21] Smith GD. A modified Valsalva manoeuvre results in greater termination of supraventricular tachycardia than standard Valsalva manoeuvre. Evid Based Med. (2016) 21(2):61. 10.1136/ebmed-2015-110357 26729773

[22] Lu Z Zhu J Gao M Song Q Pan D Huang C Efficacy and safety of modified Valsalva maneuver for treatment of paroxysmal supraventricular tachycardia: a meta-analysis. J Int Med Res. (2024) 52(1):3000605231220871. 10.1177/03000605231220871 38235710 PMC10798081

[23] Yaksh A van der Does LJ Lanters EA de Groot NM. Pharmacological therapy of tachyarrhythmias during pregnancy. Arrhythm Electrophysiol Rev. (2016) 5(1):41–4. 10.15420/aer.2016.1.2 27408722 PMC4940191

[24] Joglar JA Page RL. Antiarrhythmic drugs in pregnancy. Curr Opin Cardiol. (2001) 16(1):40–5. 10.1097/00001573-200101000-00006 11124717

[25] Fischer AJ Diller GP Uebing A Nürnberg JH Hebe J. Antiarrhythmic drugs-safety and efficacy during pregnancy. Herzschrittmacherther Elektrophysiol. (2021) 32(2):145–51. 10.1007/s00399-021-00759-2 33779803

[26] Elkayam U Goodwin TM. Adenosine therapy for supraventricular tachycardia during pregnancy. Am J Cardiol. (1995) 75(7):521–3. 10.1016/S0002-9149(99)80597-9 7864004

[27] Harrison JK Greenfield RA Wharton JM. Acute termination of supraventricular tachycardia by adenosine during pregnancy. Am Heart J. (1992) 123(5):1386–8. 10.1016/0002-8703(92)91051-2 1575160

[28] Leffler S Johnson DR. Adenosine use in pregnancy: lack of effect on fetal heart rate. Am J Emerg Med. (1992) 10(6):548–9. 10.1016/0735-6757(92)90181-V 1388381

[29] Petersen K M Jimenez-Solem E Andersen JT Petersen M Brødbæk K Køber L β-Blocker treatment during pregnancy and adverse pregnancy outcomes: a nationwide population-based cohort study. BMJ Open. (2012) 2(4):e001185. 10.1136/bmjopen-2012-001185 22815467 PMC3401834

[30] Lip GY Beevers M Churchill D Shaffer LM Beevers DG. Effect of atenolol on birth weight. Am J Cardiol. (1997) 79(10):1436–8. 10.1016/S0002-9149(97)00163-X 9165181

[31] Montan S Ingemarsson I Marsál K Sjöberg NO. Randomised controlled trial of atenolol and pindolol in human pregnancy: effects on fetal haemodynamics. Br Med J. (1992) 304(6832):946–9. 10.1136/bmj.304.6832.946 1581716 PMC1882301

[32] Rubin PC Butters L Clark DM Reynolds B Sumner DJ Steedman D Placebo-controlled trial of atenolol in treatment of pregnancy-associated hypertension. Lancet. (1983) 1(8322):431–4. 6131164

[33] Byerly WG Hartmann A Foster DE Tannenbaum AK. Verapamil in the treatment of maternal paroxysmal supraventricular tachycardia. Ann Emerg Med. (1991) 20(5):552–4. 10.1016/S0196-0644(05)81615-4 2024796

[34] Luszczki JJ Trojnar MK Trojnar MP Kimber-Trojnar Z Szostakiewicz B Zadrozniak A Effects of three calcium channel antagonists (amlodipine, diltiazem and verapamil) on the protective action of lamotrigine in the mouse maximal electroshock-induced seizure model. Pharmacol Rep. (2007) 59(6):672–82. 18195456

[35] Debreceni D Janosi K Vamos M Komocsi A Simor T Kupo P. Zero and minimal fluoroscopic approaches during ablation of supraventricular tachycardias: a systematic review and meta-analysis. Front Cardiovasc Med. (2022) 9:856145. 10.3389/fcvm.2022.856145 35479287 PMC9037593

[36] Janosi K Debreceni D Janosa B Bocz B Simor T Kupo P Visualizable vs. Standard, non-visualizable steerable sheath for pulmonary vein isolation procedures: randomized, single-centre trial. Front Cardiovasc Med. (2022) 9:1033755. 10.3389/fcvm.2022.1033755 36465461 PMC9709402

[37] Dick M 2nd Scott WA Serwer GS Bromberg BI Beekman RH Rocchini AP Acute termination of supraventricular tachyarrhythmias in children by transesophageal atrial pacing. Am J Cardiol. (1988) 61(11):925–7. 10.1016/0002-9149(88)90377-3 3354471

